# Cervical Range of Motion Analysis Performed with an Accelerometer: A Study of Intersession Reliability for Dental Practice

**DOI:** 10.3390/healthcare11101428

**Published:** 2023-05-15

**Authors:** Alessandro Nota, Laura Pittari, Laura Gamba, Francesco Manfredi Monticciolo, Alessia Lannes, Carlotta Carta, Alessandro Beraldi, Alberto Baldini, Giuseppe Marzo, Simona Tecco

**Affiliations:** 1 Dental School, Vita-Salute San Raffaele University and Department of Dentistry, IRCCS San Raffaele Hospital, 20132 Milan, Italy; nota.alessandro@hsr.it (A.N.); laura_pittari@hotmail.it (L.P.); gamba.laura@hotmail.it (L.G.); francesco.manfredi.monticciolo@gmail.com (F.M.M.); a.lannes@studenti.unisr.it (A.L.); c.carta@studenti.unisr.it (C.C.); 2 Department of Clinical Medicine, Public Health, Life and Environmental Sciences (MeSVA), University of L’Aquila, 24100 L’Aquila, Italy; giuseppe.marzo@univaq.it; 3 Private Practice, 20100 Milan, Italy; info@studioberaldi.net; 4 Private Practice, 24100 Bergamo, Italy; studiomedicobaldini@gmail.com

**Keywords:** cranio-cervical-mandibular disorders, accelerometer, range of motion, digital dentistry, temporomandibular disorders, intersession reliability

## Abstract

During the clinical examination of subjects with temporomandibular disorders (TMDs), the dentist sometimes must evaluate the cervical spine, due to the anatomical and functional connections between the cervical portion of the spine and the stomatognathic apparatus. The accelerometer is a device that evaluates the Range of Motion (ROM) of the main movements of the head on the neck. To date, only a few studies have investigated the repeatability of the use of the accelerometer in the assessment of cervical ROM. Therefore, the present longitudinal observational study analyzed the repeatability of acquired cervical movements on a sample of volunteer subjects who underwent accelerometer testing. A sample of 32 subjects was tested twice within 14 days to assess cervical ROM using a standardized protocol based on a review of existing literature. The results show that the examination is reliable for all the positions of the jaw, except for the parameters regarding the asymmetries of rotation and bending. In conclusion, the accelerometer can be considered a reliable tool for evaluating the active cervical ROM. However, further studies will be necessary to make better evaluations.

## 1. Introduction

Recent studies showed a solid functional connection between the masticatory muscular system and the cervical region [1,2]. There are connections on an anatomical, biomechanical, and neurophysiological basis between the stomatognathic system and the cervical portion of the spine, as shown by Alghadir et al., suggesting that the jaw sensory motor system can modulate postural control mechanisms. They demonstrated that gum chewing activity can enhance postural stability during upright standing on an unstable surface and in the absence of visual input in healthy young adults [3]. Eriksson et al. reported that during normal mouth opening, extension occurs at the cervical-cranial junction, and restriction in the upper cervical spine may decrease a patient’s mouth-opening capacity [4]. Furthermore, associations between temporomandibular disorders (TMDs) and strain in the musculoskeletal system of the head-neck area have been demonstrated [2,5,6,7]. As reported, patients suffering from neck pain showed a higher prevalence of TMD than the healthy population [8,9]. Liang et al. demonstrated that subjects with masticatory myofascial pain have greater neck disability than asymptomatic controls and that the greater the degree of neck disability, the greater the anterior temporalis, sternocleidomastoid and upper trapezius muscle sensitivity [10]. Furthermore, increased pain sensitivity of the cervical muscles in TMD patients and the decreased pain threshold of the masticatory muscles in patients with chronic neck pain have been observed. Thus, central sensitization was hypothesized in these patients [11,12]. The dentist often examines the cervical and sternocleidomastoid muscles through palpation in subjects affected by TMD symptomatology. In addition, there are various medical devices that allow the dentist to evaluate the cervical tract of the spine. For instance, surface electromyography (sEMG) of the muscles of the head-neck district allows the study of the action potentials generated during the contraction or the resting condition of these muscles. This assessment could be very important because a dysfunction of the stomatognathic apparatus may be associated with an alteration in the normal pattern of sEMG activity of these muscles [13].

The evaluation of the amplitude of the main movements of the head on the neck stands out as an essential part of the TMJ examination [14]. The Range of Motion (ROM) of the cervical spine is a common parameter in the assessment of cervical disorders. It consists of the active or passive measurement of the width of the angles resulting from three cervical movements (rotation, lateral inclination, and flexion-extension of the head) and comparing the results obtained with average values recorded in the healthy population or comparing the asymmetries between the two body sides [14,15,16]. Scientific literature suggests that the alterations of the cervical ROM are related to various cervical pathologies, for example cervical discectomy, fusion, spondylosis etc. [15,16,17].

The cervical ROM can be measured by various performance-based outcome measures (PBOM), such as inclinometers, measuring tapes, goniometers, visual estimates (but still with poor literature evidence) and Smartphone applications (apps) [18]; accessible, low cost and practical options.

Recently the Cervical ROM device, known as the Accelerometer in the literature, has been introduced in the dental field and successfully used for this purpose [14,19,20]. Although Ambusam et al. [21] demonstrated the excellent intra-rater reliability of an accelerometer in the analysis of the head excursion during typing tasks, there are no studies in the literature assessing the intersession reliability of cervical ROM measures with this device. This could be significant in order to ensure the trustworthiness of the exam in testing the effects of a therapy in the short or long term and monitoring the patient’s cervical changes.

The aim of the present study was to analyze the intersession repeatability of the use of the accelerometer in the assessment of cervical ROM.

## 2. Materials and Methods

A total of 23 subjects (16 F, 7 M; mean age 28.26 ± 8.94 years) were enrolled in the study between January 2023 and April 2023 and analyzed at the Department of Dentistry of IRCCS San Raffaele Hospital in Milan (Italy).

All participants were over 18 years of age and free from spontaneous pain in the cervical spine.

Patients with systemic diseases affecting the mobility of the cervical spine (such as scoliosis, fractures, trauma, morphological alterations, degenerative diseases), subjects affected by TMDs according to DC/TMD [22], subjects who have suffered craniosacral trauma, subjects undergoing craniosacral physiotherapy treatments, subjects undergoing orthodontic and dental treatments, subjects unable to attend the two sessions 14 days apart were all excluded from this study.

Volunteers signed a consent form after being fully informed about the nature of the study. Furthermore, the study was approved by the ethic committee of IRCCS San Raffaele Hospital (Milan, Italy) with the document “parere09/int/2023”. Subsequently, the subjects underwent an anamnestic and clinical examination in order to assess mandibular parameters according to DC/TMD [22].

### Instrumentation, Procedure and Statistical Analysis

**Figure 1:**
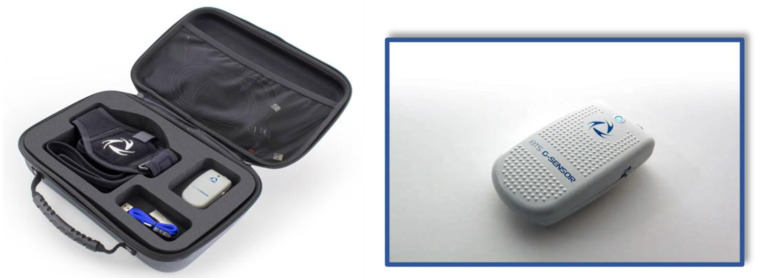
Baiobit Accelerometer.

**Figure 2:**
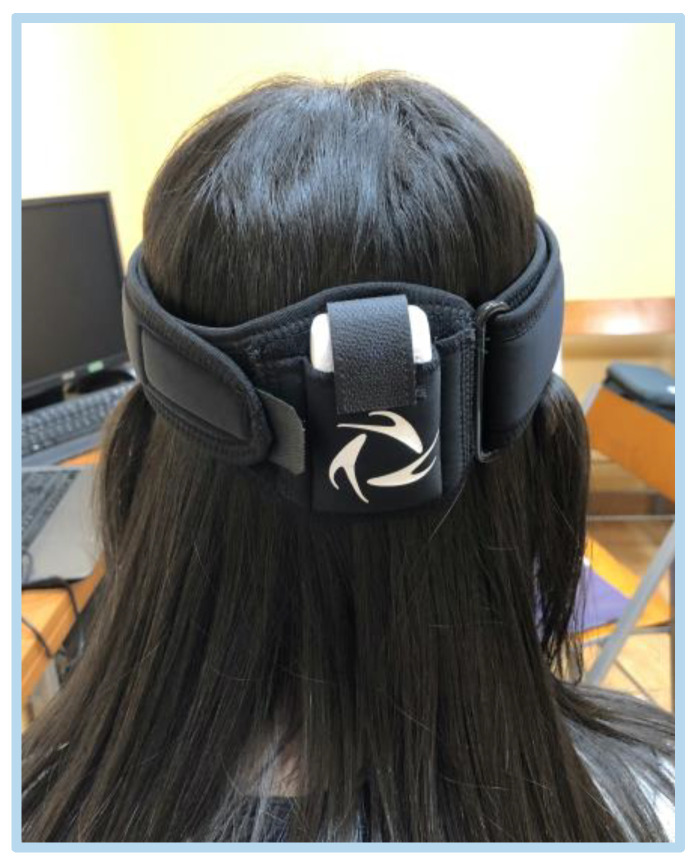
Accelerometer positioned.

To assess cervical ROM, an accelerometer (or inertial sensor) (Baiobit, Rivelo S.r.l., Garbagnate Milanese, Italy) was used (Figure 1 and Figure 2). This accelerometer is a light and handy medical device designed to make precise and fast measurements. The kit consists of one wearable motion sensor that guarantees freedom of movement and three belts of various lengths fit for different body areas. The wireless sensor consists of inertial MEMS platforms (Micro Electro-Mechanical Systems) each one composed of a triaxial accelerometer, triaxial gyroscope and triaxial magnetometer but it will be called “accelerometer” in the text even if all the three components are fundamental for the analyses performed. The information provided by gyroscope accelerometers and magnetometers is combined by Sensor Fusion algorithms and sent to the software via Bluetooth connection. The sensor captures and transmits data to the PC for automatic report processing and creation. The software using the data of the sensors calculates the amplitude angles for each movement of the cervical range of motion.

Each subject underwent two exam sessions, with a 14-day break. The two sessions were conducted in the same way and under conditions as identical as possible, to allow the study of intersection reliability. To exclude the potential inter-observer variability, all tests were performed by the same operator, and before each test, the software was programmed to perform an automatic calibration in order to exclude potential systematic error (able to influence the intrasession reliability).

Each volunteer was required to comply with specific requirements: to not undertake new sports training programs involving the head-neck district in the 15 days preceding the test; to not take topical and/or systemic pain medication within 15 days prior to the test; to not undergo physical stress in the hours preceding the test; to not engage in sports activities during the hours preceding the test; to not consume alcohol during the hours preceding the test.

Before the sessions, both T0 and T1, a questionnaire was submitted to volunteers to ensure that all the above requirements had been met. If the subject had not complied with even one of the indications, the session was canceled and rescheduled to ensure that the test would be carried out according to the protocol. In case of cancellation of the T1 session the T0 would have to be repeated in order to provide the 14-day break between T0 and T1, as per protocol.

To measure the cervical ROM the accelerometer was placed using the elastic band provided by the manufacturer, at the back of the head (precisely at the protrusion of the occipital bone). Subjects sat upright in the chair with their heads parallel to the Frankfurt horizontal plane and their backs supported by the chair.

Subjects were required to execute the following movements:

- right/left rotation of the head: the subject rotates the head to the right and left as much as possible trying to keep the chin parallel to the shoulders.
- bending and extension of the head: the subject bends and extends the head as much as possible.
- right/left lateral bending of the head: the subject, looking straight ahead, bends the head sideways to the right and left, as much as possible, trying to bring the ear toward the shoulder.

Other movements (involving the thoracic spine and shoulders) were avoided by subjects with the help of an operator behind them.

Subjects were informed on how to perform the exercises correctly. Subjects were required to perform only one movement per exercise. After a test, effective measurement of each movement was made using the 9-axis accelerometer Baiobit (Rivelo S.r.l) in five different mandibular positions in random order; the mandibular positions were numbered 1 to 5 and then randomly extracted before each test.

**Figure 3:**
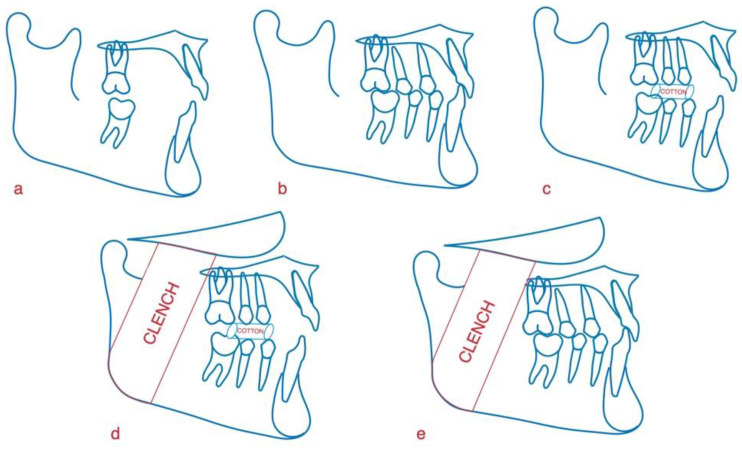
(**a**) mandibular rest position (Rest); (**b**) maximum intercuspation (Max. Int); (**c**) mandibular position with cotton rolls (Cotton); (**d**) clenching on cotton rolls (Cle. Cot); (**e**) clenching in maximum intercuspation (Clenching).

The following mandibular positions were tested (Figure 3): Rest: habitual position of rest (no teeth contact); Max. Int.: maximum intercuspation (light contact between teeth); Cotton: mandibular position with cotton rolls (8 mm thick and 37 mm long) placed distally to the canines between the dental arches; Cle. Cot: clenching on cotton rolls; Clenching: maximum intercuspation with clenching.

For achieving the positions Cotton and Cle. Cot, two cotton rolls (8 mm thick and 37 mm long) were placed between the dental arches distally to the canines.

In case the operator observed that the movement had been performed incorrectly, the subject was required to repeat the exercise.

The following parameters were analyzed:

- Rot. left (left rotation)
- Rot. right (right rotation)
- Asy R (difference between right and left rotation angles)
- Ext (extension)
- Flex (flexion)
- Ben. sx (left side bending)
- Ben. right (right side bending)
- Asy B (difference between right and left lateral bending angles)

With bending/extension being two different movements with different physiological amplitudes, the parameter of the bending/extension asymmetry cannot be calculated.

Data expressed in degrees were exported from the software. A normal data distribution was ensured by a Shapiro-Wilk test. Statistical mean and standard deviation were calculated for descriptive analysis. A Student’s t-test for paired data analysis was applied to perform a comparison between sessions (T0 and T1). A significance level of 0.05 was adopted.

The present study aimed to assess the reliability of the cervical ROM test with an accelerometer and to verify the reproducibility of the measurements between sessions.

The intraclass correlation coefficient (ICC) was estimated to ensure the reliability of the test between sessions, since it was possible to believe that no systematic error occurred [17].

The ICC is a number between 0 and 1 used to measure the reliability. An ICC of more than 0.75 indicates good to excellent reliability, an ICC of between 0.40 and 0.75 indicates discrete to good reliability, and an ICC of less than 0.40 indicates poor reliability [18].

According to this, the sample size was established by performing an a-priori sample size analysis in order to achieve a sample power of 80% and a significance of 0.05 with a minimum acceptable ICC of 0.40, confirming that a minimum of 19 subjects must be included in the study.

## 3. Results

A total of 23 subjects (16 F, 7 M; mean age 28.26 ± 8.94 years; mean weight 61.52 ± 13.17; mean height 169.61 ± 7.80 cm) were included in this study and were tested twice for cervical mobility within 14 days.

Table 1, Table 2, Table 3, Table 4 and Table 5 summarize the descriptive statistics of the results, describing the different methods of analysis with different mandibular positions (rest, maximum intercuspation, rest with cottons, clenching cottons and clench); as we can see from the *p*-values, in general, the difference in time between the trials had no impact on the recording of the mandibular position, confirming the reliability of measurements. A significant difference between the sessions was only observed for the bending degrees of the head measured at maximum intercuspation (Table 4).

Repeatability was studied by estimating the ICC for each of the five mandibular positions analyzed. Table 6 summarizes the ICC values. All values exceed the threshold of 0.40 indicating good reliability, with many values exceeding 0.75 indicating, therefore, good reliability and, in some cases, excellent. Unlike the other parameters, the data showed poor repeatability in the assessment of the magnitude of asymmetry (Asy R and Asy B).

## 4. Discussion

The present study aimed to investigate the intersession reliability of the cervical ROM exam conducted with an accelerometer in different mandibular positions. As the statistical comparison between the two observations showed the absence of statistical significance, the ICC results seem to demonstrate that the cervical ROM exam has good intersession reliability in all the mandibular positions (rest position, maximum intercuspation, mandibular position with cotton rolls, clenching with cotton rolls) analyzed in both sessions (T0 and T1).

Consequently, the use of the 9-axis accelerometer to assess cervical ROM with a standardized protocol applied in different sessions was found to be a repeatable examination with important implications in clinical practice, as it should be possible to verify the effects of a short or long-term therapeutic plan by monitoring the changes in the cervical spine.

Poor repeatability was observed in the assessment of the asymmetry of rotation and lateral bending (Asy R and Asy B) and although the identification of the presence of an asymmetry in the range of movement represents a negative sign in terms of function, the magnitude of the asymmetry cannot be considered a reliable value when compared over time (or before and after treatment) as significant changes in the parameter may occur due to other factors [3,14,19,20,21,23,24,25]. The present study is the first that analyzes the intersession reliability of an accelerometer in the analysis of the cervical ROM, and the repeatability of accelerometers for the calculation of cervical ROM represents a promising and constantly evolving topic. Pérez Fernández et al. [25] reported ICC values that indicate excellent intra and inter-rater repeatability of the assessment of the ROM flexor skull-cervical using a wearable inertial sensor. The study was carried out on a sample of asymptomatic subjects. This sample underwent a craniocervical flexion test (CCFT) that specifically evaluates the function of deep cervical flexor muscles. Another work [19] focused on the repeatability of inertial sensors, extending the measurement of the ROM of adjacent segments covering the entire spine. Also, in this case, high ICC values were detected, demonstrating the good reliability of these electromedical devices for measuring spinal ROM.

In addition to “pure” inertial systems, other devices, such as smartphones, can currently be used for the evaluation of cervical ROM with some limitations compared to our work. Quek et al. [20] showed that an Android phone can be a valuable and reliable tool for measuring the ROM of cervical flexion, extension, and lateral bending, but not for cervical rotation. The results of the cervical rotation were not considered reliable as, in the tested position, both sagittal and frontal measurements were based on gravity-dependent accelerometers within the phone, but movements in the transverse plane were detected by the magnetometer, which can be affected by any surrounding magnetic field.

The presence of multiple studies regarding the repeatability of different TMJ evaluation instruments highlights the need for dentists to have daily access to devices that have good reliability in their detections [26]. As previously mentioned, the stomatognathic apparatus and the cervical spine have deep interconnections [4,5,27,28,29,30,31]. For this reason, even if the present study analyzed the cervical ROM on subjects free from TMDs, the analysis of the cervical ROM in dentistry is more useful in subjects with TMDs [28,29]. In fact, previous studies showed that the presence of TMDs could affect the cervical ROM [26] and that, even if in healthy subjects there is no influence of the mandibular posture on the active cervical ROM [32], in subjects with TMDs the bite position affects the isometric strength of cervical flexors [33,34]. Consequently, future studies should focus on analyzing the intersession reliability of this exam in subjects with TMDs.

Considering that measuring the range of motion of the cervical spine is common in assessing spinal and cervical disorders [35], the use of the accelerometer provides the possibility of having objective reports of high clinical utility that can be shared between clinicians to ensure a multidisciplinary approach on craniocervical-mandibular disorders [36].

Scientific literature has proven that a multi-disciplinary approach can be extended in dealing with these patients, not just to the gnathologist, but to the physiatrist, physiotherapist, and other specialists.

The present study has some limits, among them is the young age of the participants that can reduce the application of the results to a wider population, furthermore subjects with TMDs should also be evaluated in future studies [37,38,39].

It should be observed that even if the sample is not homogeneous for sex and age, and even if this seems to have a small impact of the cervical ROM starting from the age of 30 years [40], the analyses of the present studies were performed within the same group, comparing the cervical ROM of the same subjects between the two sessions. Consequently, the analyses should not be influenced by the homogeneity of the sample.

## 5. Conclusions

The 9-axis accelerometer seems to be a reliable device for evaluating the active range of movement of the cervical spine over a period of time.

Asymmetry of rotation and lateral bending parameters, due to poor reliability, were considered unreliable in comparing parameters between different sessions.

## Figures and Tables

**Table 1:** Descriptive statistics and significance of data recorded in the mandibular resting position.

Rest				
		T0	T1	*p*-Value
L Rot.	Mean	74.13	74.13	1
	Sd	8.71	10.57	
R Rot.	Mean	72.63	70.44	0.07
	Sd	9.03	8.81	
Asy R	Mean	7.13	7.13	1
	Sd	5.06	6.66	
Ext	Mean	63.09	62.03	0.49
	Sd	15.16	16.86	
Flex	Mean	59.34	58.78	0.75
	Sd	11.32	13.45	
L Ben.	Mean	43.13	41.25	0.09
	Sd	7.85	8.97	
R Ben.	Mean	41.03	40.34	0.49
	Sd	7.68	7.15	
Asy B	Mean	5.78	4.22	0.07
	Sd	4.48	3.35	

L Rot: Left Rotation; R Rot: Right Rotation; Asy R: Rotation Asymmetry; Ext: Extension; Flex: Flexion; L Ben: Left Bending; R Ben: Right Bending; Asy B: Bending Asymmetry.

**Table 2:** Descriptive statistics and significance of data recorded at maximum intercuspation.

Max. Int				
		T0	T1	*p*-Value
L Rot.	Mean	73.84	74.09	0.85
	Sd	8.11	8.73	
R Rot.	Mean	71.59	71.16	0.8
	Sd	9.6	10.52	
Asy R	Mean	6.31	7.31	0.51
	Sd	5.73	7.78	
Ext	Mean	62.28	60.13	0.18
	Sd	14.06	16.33	
Flex	Mean	59.28	55.81	0.03
	Sd	10.46	11.85	
L Ben.	Mean	41.59	41.91	0.81
	Sd	7.29	8.92	
R Ben.	Mean	39.06	40.25	0.25
	Sd	9.34	8.31	
Asy B	Mean	6.72	5.53	0.17
	Sd	4.37	4.44	

L Rot: Left Rotation; R Rot: Right Rotation; Asy R: Rotation Asymmetry; Ext: Extension; Flex: Flexion; L Ben: Left Bending; R Ben: Right Bending; Asy B: Bending Asymmetry.

**Table 3:** Descriptive statistics and significance of data recorded in the mandibular position with cotton rolls.

Cottons				
		T0	T1	*p*-Value
L Rot.	Mean	73.56	73.41	0.91
	Sd	9.08	10.53	
R Rot.	Mean	71.75	71.34	0.72
	Sd	8.71	9.57	
Asy R	Mean	6.81	7.25	0.75
	Sd	5.2	7.32	
Ext	Mean	59.41	58.56	0.68
	Sd	16.1	17.92	
Flex	Mean	59.66	57.5	0.35
	Sd	11.09	12.39	
L Ben.	Mean	41.22	41.97	0.54
	Sd	6.88	8.86	
R Ben.	Mean	40.34	39.94	0.67
	Sd	8.21	8.93	
Asy B	Mean	5.63	4.84	0.45
	Sd	4.23	4.23	

L Rot: Left Rotation; R Rot: Right Rotation; Asy R: Rotation Asymmetry; Ext: Extension; Flex: Flexion; L Ben: Left Bending; R Ben: Right Bending; Asy B: Bending Asymmetry.

**Table 4:** Descriptive statistics and significance of data recorded clenching in mandibular position with cotton rolls.

Cle. Cot				
		T0	T1	*p*-Value
L Rot.	Mean	75.03	73.63	0.3
	Sd	6.98	9.79	
R Rot.	Mean	72.84	71.97	0.49
	Sd	10.08	8.47	
Asy R	Mean	6.19	6.16	0.98
	Sd	4.96	4.54	
Ext	Mean	60.47	59.59	0.65
	Sd	13.81	13.76	
Flex	Mean	62.5	60.44	0.13
	Sd	9.42	11.47	
L Ben.	Mean	42.5	41.66	0.53
	Sd	6.43	9.13	
R Ben.	Mean	41.09	41.09	1
	Sd	7.52	7.68	
Asy B	Mean	4.59	5.13	0.55
	Sd	4.08	4.27	

L Rot: Left Rotation; R Rot: Right Rotation; Asy R: Rotation Asymmetry; Ext: Extension; Flex: Flexion; L Ben: Left Bending; R Ben: Right Bending; Asy B: Bending Asymmetry.

**Table 5:** Descriptive statistics and significance of data recorded clenching in the maximum intercuspation position.

Clenching				
		T0	T1	*p*-Value
L Rot.	Mean	74.06	73.63	0.76
	Sd	8.97	10.13	
R Rot.	Mean	72.47	72.28	0.89
	Sd	11.44	9.46	
Asy R	Mean	9.41	6.53	0.08
	Sd	7.54	4.56	
Ext	Mean	62.44	60.84	0.35
	Sd	14.99	17.63	
Flex	Mean	61.75	57.94	0.04
	Sd	12.79	14.19	
L Ben	Mean	40.91	41.75	0.52
	Sd	7.38	10.07	
R Ben	Mean	40.03	40.81	0.52
	Sd	7.89	8.23	
Asy B	Mean	5	6.56	0.1
	Sd	3.06	4.79	

L Rot: Left Rotation; R Rot: Right Rotation; Asy R: Rotation Asymmetry; Ext: Extension; Flex: Flexion; L Ben: Left Bending; R Ben: Right Bending; Asy B: Bending Asymmetry.

**Table 6:** ICC values in the five mandibular positions.

Mandibular Pos.	Rest	Max. Int	Cottons	Cle. Cot	Clenching
L Rot.	0.66	0.65	0.73	0.66	0.66
R Rot.	0.74	0.56	0.77	0.73	0.74
Asy R	0.04	0.26	0.29	0.14	0.02
Ext	0.86	0.85	0.79	0.69	0.85
Flex	0.71	0.73	0.42	0.76	0.72
L Ben.	0.76	0.60	0.67	0.60	0.70
R Ben.	0.73	0.80	0.82	0.84	0.65
Asy B	0.32	0.42	0.08	0.31	0.22

L Rot: Left Rotation; R Rot: Right Rotation; Asy R: Rotation Asymmetry; Ext: Extension; Flex: Flexion; L Ben: Left Bending; R Ben: Right Bending; Asy B: Bending Asymmetry.

## Data Availability

Individual patients’ data are not showed for privacy reasons, but are available upon reasonable request at Vita-Salute San Raffaele University in Milan, Italy.
